# Blind deconvolution in autocorrelation inversion for multiview light‐sheet microscopy

**DOI:** 10.1002/jemt.24085

**Published:** 2022-02-24

**Authors:** Elena Corbetta, Alessia Candeo, Andrea Bassi, Daniele Ancora

**Affiliations:** ^1^ Politecnico di Milano, Department of Physics piazza Leonardo da Vinci 32 Milan Italy; ^2^ National Council of Research of Italy Institute of Photonics and Nanotechnology Milan Italy

## Abstract

Combining the information coming from multiview acquisitions is a problem of great interest in light‐sheet microscopy. Aligning the views and increasing the resolution of their fusion can be challenging, especially if the setup is not fully calibrated. Here, we tackle these issues by proposing a new reconstruction method based on autocorrelation inversion that avoids alignment procedures. On top of this, we add a blind deconvolution step to improve the resolution of the final reconstruction. Our method permits us to achieve inherently aligned, highly resolved reconstructions while, at the same time, estimating the unknown point‐spread function of the system.

**Research Highlights:**

- We tackle the problem of multiview light‐sheet deconvolution with a blind approach of autocorrelation inversion
- Our method recovers the object and PSF, requires no alignment and calibration, and enhances the reconstruction of the specimen.

## Graphical abstract

In this publication, we propose a new method for image reconstruction in light‐sheet microscopy based on multi‐view acquisitions. While reconstructing, our algorithm also estimates the point‐spread function of the microscope, increasing the resolution of the reconstructed image simultaneously. This protocol does not need initial alignment of the dataset and starts with a blind estimation of the PSF, rendering it a flexible solution for automatic high‐resolution reconstructions. The image shows a synthetic multi‐view sample, blindly deconvolved with the proposed inversion scheme: blurred and noisy object (top‐left triangle) and final reconstruction (bottom‐right triangle).

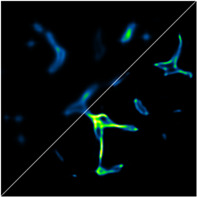

## 1 INTRODUCTION

Image formation in microscopy can be described mathematically by the convolution of the perfectly resolved object with the microscope's point spread function (PSF) (Mertz, 2019). The latter represents the impulse response of the system and, ideally, should be as narrow as possible to obtain the best image resolution. When the PSF diverges from the ideal delta function, image‐restoration techniques may be used to improve the three‐dimensional image quality. Among others, deconvolution (the process of inverting the convolution) aims to retrieve a sharper estimation of the object, reducing optical blur and noise by reassigning the energy distribution of the signal according to the PSF.

In Light Sheet Fluorescence Microscopy (LSFM) (Huisken & Stainier, 2009; Olarte et al., 2018), the illumination and detection optical paths are independent and orthogonal to each other: the PSF depends on both the illumination and detection optics and, typically, results in a function that is more elongated along the detection axis. For this reason, multi‐view acquisitions are frequently adopted as a method to obtain isotropic resolution (Calisesi et al., 2020; Swoger et al., 2007). Moreover, in the presence of slightly absorbing diffusive specimens (Rieckher et al., 2018), multiview acquisition facilitates the reconstruction of the entire imaged volume by accessing favorable views of the object. However, preprocessing steps are necessary to align and fuse all the acquisitions. In a typical multi‐view LSFM pipeline, stacks of images are acquired from various angles, they are rotated, aligned, and combined together. Each of these steps requires dedicated care. The alignment procedure involves finding the rigid shift that guarantees the best overlaps of each view against each other. In general, the fusion can be performed by computing simple averages or with more advanced methods, for example, by considering Poisson statistics (Swoger et al., 2007) or optimized Bayesian strategies (Fernandez et al., 2010; Guo et al., 2020; Preibisch et al., 2014).

To complicate the reconstruction further, the knowledge of the PSF is not always accessible (Kundur & Hatzinakos, 1996). In a light sheet microscope, the acquisition of the PSF is performed by acquiring beads placed in the imaging chamber. However, this procedure may be impractical: this is the case in some clearing media (Ueda et al., 2020) where it is difficult to prepare beads phantoms, or in fluidic‐based systems (Sala et al., 2020). In these cases, blind deconvolution is the only possible approach to increase imaging resolution (Kundur & Hatzinakos, 1996).

To solve the issues of alignment and fusion, we have proposed the Anchor Update (AU) algorithm (Ancora & Bassi, 2021). The protocol works out a deconvolved reconstruction from the autocorrelation estimation, exploiting the fact that working in the shift space guarantees implicit alignment. This implies that the solution of the autocorrelation problem always returns reconstructions that are aligned intrinsically at sub‐pixel resolution [14].

In the present manuscript, we describe a strategy for tomographic reconstruction in multi‐view acquisitions corrupted by unknown blurring, that can be utilized in those cases when the PSF is not easily measurable. To this end, we design a blind deconvolution approach that acts in the autocorrelation space. This permits the formation of inherently aligned reconstructions, increasing the resolution simultaneously without any prior knowledge of the PSF of the system. We take inspiration from the Richardson‐Lucy deconvolution (RLD) (Lucy, 1974; Richardson, 1972) and its blind implementation (Fish et al., 1995), extending the blind approach to the autocorrelation domain. First, we introduce the computational methods by validating the protocol thanks to the reconstruction of synthetic samples. Here, generated images for the object and PSF are used as ground truth to assess the convergence of our algorithm. Once validated, we test our reconstruction method on real data acquired by a custom LSFM setup, with the aim of retrieving both the PSF and the aligned reconstruction. Lastly, we discuss the potential of our method in the field of image processing for optical microscopy applications.

## 2 RESULTS AND DISCUSSION

### 2.1 Working principle of blind deconvolution

The goal of any blind deconvolution algorithm is to obtain a deblurred version of the original image without any knowledge of the response of the optical system used for image acquisition. It requires solving the deconvolution problem, $o_{\mu }=o*h$, recovering o, the original object, from the blurred measurement $o_{\mu }$ without knowing the blurring function h. There are infinite couples of images and PSFs that may reproduce the observed blurred output; thus, it may appear as a seemingly impossible problem to solve. It has been shown, however, that meaningful solutions are achievable by having weak, or even null, knowledge of the object under study and of the PSF (Kundur & Hatzinakos, 1996).

When the PSF is unknown, one of the most effective strategies is provided by a modified blind RLD method, which acts alternatively in the object and PSF domain. This protocol, introduced by Fish et al. (Rieckher et al., 2018), consists in dividing the deconvolution process into two steps, in which the object and the PSF commute their roles. First, it executes a certain number of RLD iterations to improve the object reconstruction by using a fixed guess for the PSF. Then, the PSF estimation is improved by using the previously reconstructed object as the kernel of another RLD. This cycle is repeated several times until reaching optimal reconstructions.

Since we are interested in aligned multi‐view measurements, we propose an extension of this blind method to the autocorrelation space. This approach is aimed at treating alignment and blind deconvolution together. As previously demonstrated by Ancora et al. (2021) in the case of known blurring, the AU algorithm inverts the autocorrelation of the measurement, obtaining an aligned and deconvolved reconstruction $o_{\mathrm{rec}}$. In this case, the problem that we try to solve is the inversion of the average autocorrelation, which we call $\chi_{\mu }$, estimated from the measured views:

$$\chi_{\mu }=\frac{1}{m}\sum_{i=1}^{m}o_{\mu }^{\left(i\right)}⋆o_{\mu }^{\left(i\right)}$$

In the previous equation, we have denoted the autocorrelation operator with the symbol ⋆ and with m the total number of acquired views. In practice, the problem that we tackle is separating the object from the PSF given the average autocorrelation $\chi_{\mu }=\left(o_{\mathrm{rec}}⋆o_{\mathrm{rec}}\right)*H$, where H=h⋆h is the autocorrelation of the PSF. In this representation, $o_{\mathrm{rec}}$ is the average of the aligned and deconvolved views $o^{\left(i\right)}$, and h represents the PSF that blurs the fused multiview data, computed as the average of the PSFs of each view. In a multiview setting, the PSFs simply differ by a rotation angle. Here, we aim to accomplish the same result without having any information about the microscope's PSF, h.

**FIGURE 1:**
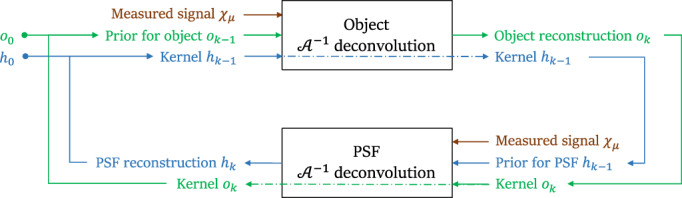
Schematics of the blind‐AU algorithm. The algorithm starts receiving as input two initial guesses, one for the object and the other for the PSF. In the first step, the autocorrelated signal $\chi_{\mu }$ is deconvolved and deautocorrelated to improve the object estimation $o_{k}$. After that, the same signal is used to restore the PSF, maintaining $o_{k}$ unchanged. Upon completion of the outer cycle indicated by arrows, the estimations $o_{k}$ and $h_{k}$ are retrieved, and the process iterated. We choose the number of iterations for each step and the number of times the outer cycle is performed depending on the experimental or simulated conditions

We address the problem as schematized in Figure 1, implementing the strategy via the iterated execution of two consecutive blocks. We keep the PSF fixed, and we reconstruct the object within the first block. We do the opposite for the second block, fixing the previously obtained object while focusing the reconstruction on the PSF. For the sake of clarity, here, we omit the index k that counts the iterations of the blocks. We start from the autocorrelation of the measurement $\chi_{\mu }$ by providing two initial guesses: one for the object, $o_{0}$, and another for the PSF, $h_{0}$. We begin by executing the AU algorithm to recover an object from the blurred autocorrelation (Ancora & Bassi, 2021) as if we knew the PSF (upper block of Figure 1). At each iteration step t, the AU algorithm calculates an intermediate kernel that contains the previous reconstruction of the object and the autocorrelation of the PSF, as in $K^{t}=o^{t}⋆H$. The kernel is defined so that its convolution with the reconstructed object matches the measured autocorrelation, $\chi_{\mu }=o_{\mathrm{rec}}*K$, once reaching the reconstruction $o_{\mathrm{rec}}$. Introducing this quantity makes the AU fixed‐point update described in Equation (1) similar to the RL deconvolution. After a certain number of iterations, we reverse the role of object and PSF inside the iterative process: we focus on reconstructing the PSF while keeping the object fixed as if we knew it (bottom block of Figure 1). The process is formalized in Equation (2). This equation is similar to Equation (1), except for the introduction of a different kernel $K^{t^{\prime }}={h^{t}}^{\prime }⋆O$. Now, K takes the current PSF and the autocorrelation O=o⋆o of the previously reconstructed object. We cycle through these AU blocks several times until obtaining accurate reconstructions for the PSF and object. Since we assume that we have no information about the PSF, we will refer to this strategy as the Blind‐AU reconstruction protocol. By denoting with the indexes t and t′ the iterations within AU cycles, and with the subscript *k* the steps that run through the AU‐blocks, formally, the Blind‐AU can be written as:

(1) $$\left\{\begin{array}{c} K_{k}^{t}=o_{k}^{t}⋆H_{k-1}\;\\ \;\\ o_{k}^{t+1}=o_{k}^{t}\left[\left(\frac{\chi_{\mu }}{o_{k}^{t}*K_{k}^{t}}\right)*{\tilde{K}}_{k}^{t}\right] \end{array}\right.$$

(2) $$\left\{\begin{array}{c} K_{k}^{t^{\prime }}=h_{k}^{t^{\prime }}⋆O_{k}\;\\ \;\\ h_{k}^{t^{\prime }+1}=h_{k}^{t^{\prime }}\left[\left(\frac{\chi_{\mu }}{h_{k}^{t^{\prime }}*K_{k}^{t^{\prime }}}\right)*{\tilde{K}}_{k}^{t^{\prime }}\right] \end{array}\right.$$

As previously mentioned, the object and the PSF are denoted with o and h, whereas we indicate their autocorrelation with the uppercase letters O and H. Here, the tilde specifies that the element is expressed with reversed coordinates: $\tilde{K}=K\left(-x\right)$. The code for blind deconvolution is available on GitHub (Ancora & Corbetta, 2021).

In the following section, we test our new Blind‐AU method to reconstruct a synthetic sample, mimicking a misaligned acquisition with differently orientated point spread functions.

### 2.2 Blind PSF and object reconstructions of synthetic data

**FIGURE 2:**
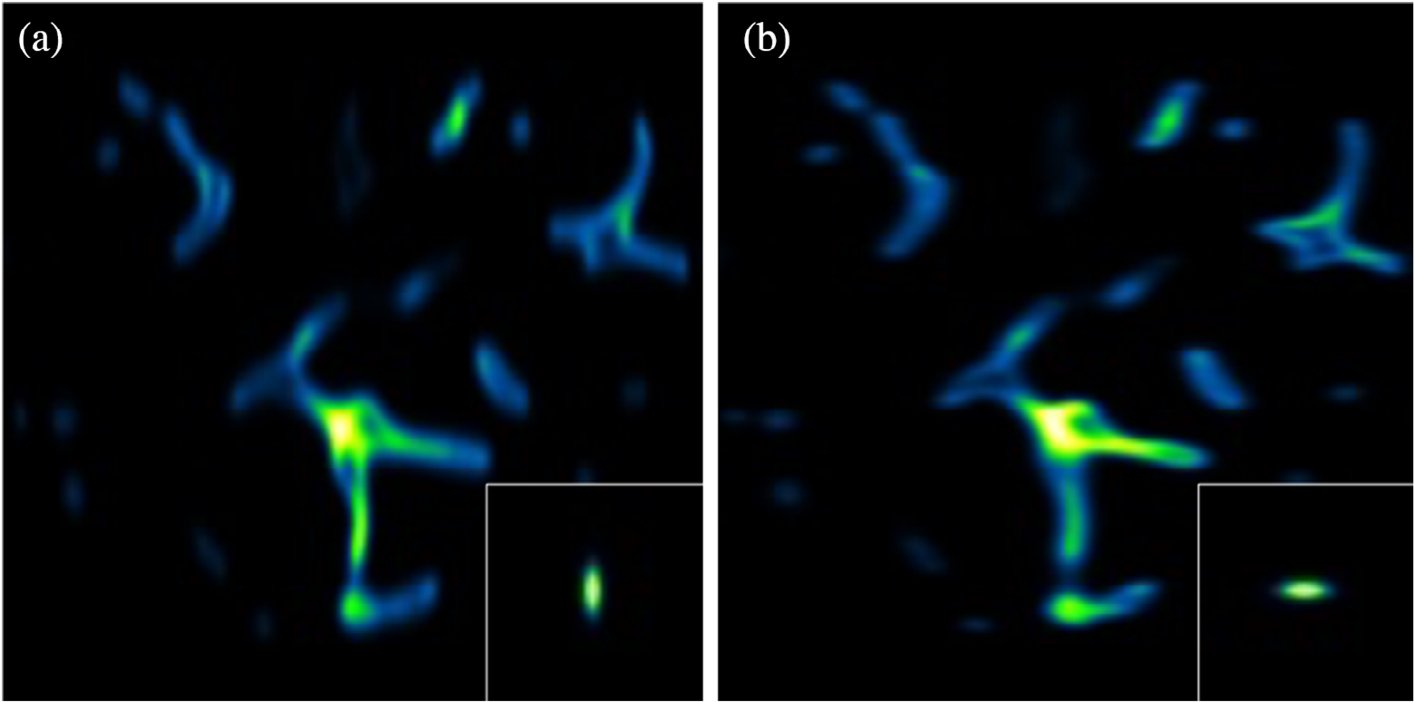
Synthetic sample for blind deconvolution. Blurred and noisy orthogonal views generated starting from an image simulating blood vessels. (a) Synthetic measurement of the ground truth image corrupted by the PSF represented in the inset. (b) Orthogonal measurement with its corresponding PSF

In multi‐view light‐sheet microscopy, the sample is observed from different angles and always exhibits a point spread function elongated along the detection axis (Swoger et al., 2007). In Figure 2, we simulate a typical tomographic section of a sample measured in an LSFM setup. For our case study, we consider a generic sample made by a random arrangement of vessel‐like structures, blurred by two PSFs elongated along the vertical (Figure 2a) and horizontal (Figure 2b) directions. These directions would correspond to the direction of the mechanical scanning of the sample through the light sheet.

**FIGURE 3:**
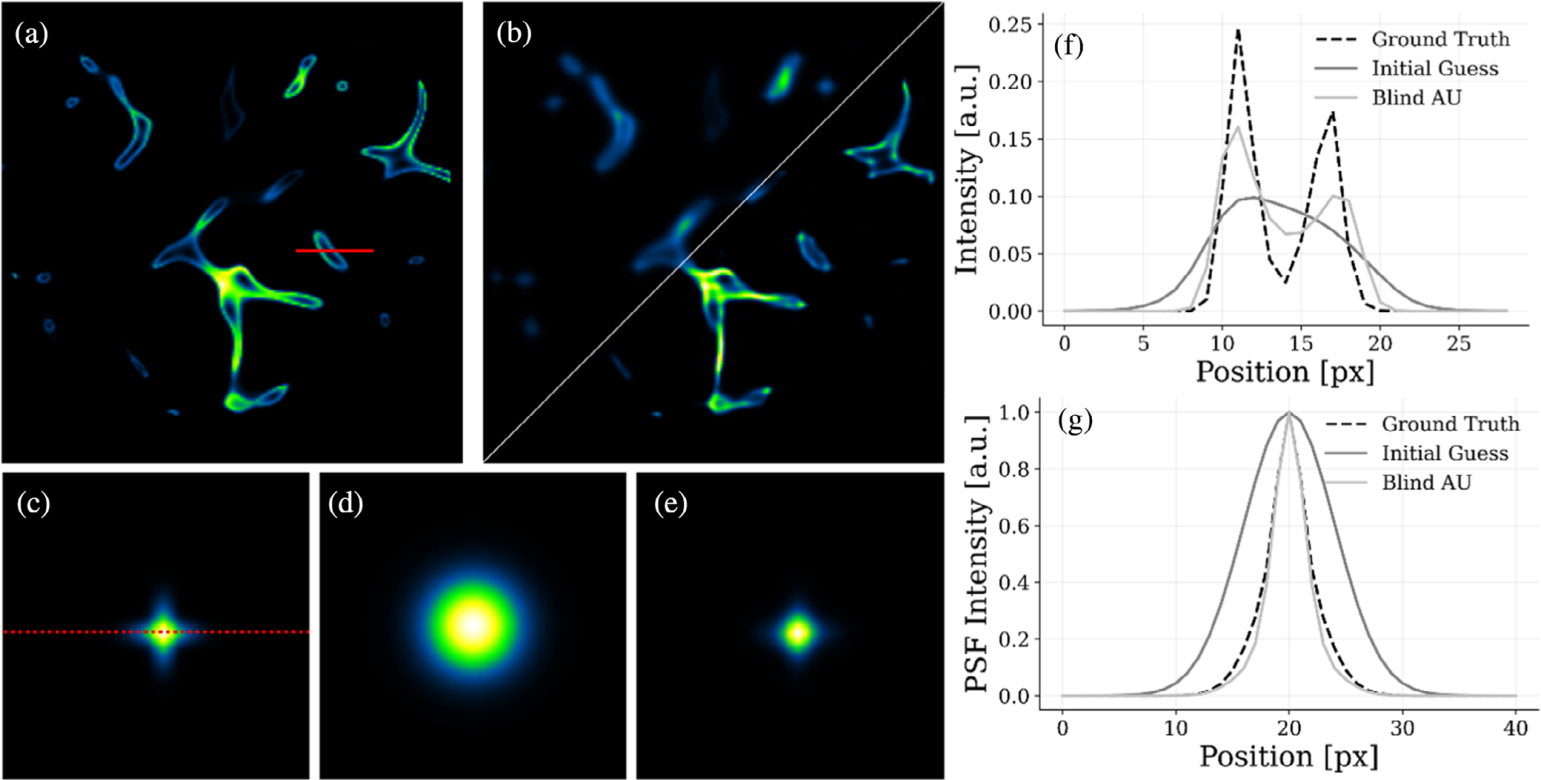
Simulated blind‐AU reconstruction. (a) Original synthetic sample resembling a vessel network. (b) Upper‐left triangle: Blurred reconstruction of synthetic data obtained by averaging orthogonal views of the object. Bottom‐right triangle: Reconstruction obtained with our blind‐AU strategy. (c) Blurring kernel resulting from the fusion of orthogonal views. (d) Initial guess for the blurring kernel from which we start our blind reconstruction. (e) PSF kernel obtained at the end of the blind‐AU algorithm. (f) Intensity profiles along the red line drawn in panel (a) of the ground truth, simulated measurement, and blind‐AU result. (g) PSF profiles along the red dashed line drawn in panel c: Blurring kernel computed as the average of the PSFs from multiple angles, initial guess for the PSF, and PSF reconstructed at the end of the blind‐AU. The software used to generate this figure is available on GitHub (Ancora & Corbetta, 2021)

We test our image reconstruction method with this dataset, aiming at obtaining a tomographic slice sharper than the mere average of both images. The original section of the synthetic sample, which is our ground truth, is shown in Figure 3a. Aligning and fusing the orthogonal views leads to the blurred reconstruction in Figure 3b (top‐left triangle). The alignment was accomplished by locating the peak of their cross‐correlation and compensating for its opposite shift (Guizar‐Sicairos et al., 2008). As a result of the fusion, the blurred reconstruction is affected by the combination of the two orthogonal PSFs that generate a star‐like point spread function (Figure 3c).

Our Blind‐AU method allows for the reconstruction of both the object, sharper than each single view, and the PSF. Since our algorithm assumes that we do not have any information about the system, we set the initial guess of the PSF to be an isotropic Gaussian with σ=4px (Figure 3d). This initialization is larger than the ground truth PSF in both directions. On the other hand, we keep the aligned average as initial guess for the object. Ideally, our protocol will adjust the guess of the PSF to the actual PSF that blurred the synthetic measurements.

We run a total of $2\times 10^{5}$ AU iterations to obtain the reconstructions: the protocols are executed in steps of 50 iterations for both the object and the PSF, and the outer blind cycle is repeated 2000 times. With this choice, the object and the PSF are processed for $10^{5}$ iterations each. The result of the object reconstruction is shown in the bottom‐right triangle of Figure 3b. The reconstructed image exhibits higher contrast and enhanced void regions, even when not directly discernible in the blurred version. We use intensity profiles to quantify the resolution increase by comparing the ground truth, the initial guess, and the de‐autocorrelated and deconvolved image (Figure 3f). We observe the profiles along the red line drawn in Figure 3a. Starting from a hump, in which the opposite walls are indistinguishable, the two peaks of the original image are recovered. The deconvolution process transfers the signal energy from the center to the two opposite lobes. Moreover, the recovered PSF reaches a remarkable result: if we start from an isotropic Gaussian function, the kernel quickly converges toward a star‐like shape (Figure 3g). For completeness, we report that the cross takes form after a few hundred iterations, and its overall shape continues to refine during the following steps.

The image quality of the reconstruction and the convergence trend of the algorithm can be quantified with the metrics defined in Section 4. For the sake of comparison, the synthetic measurement has been deconvolved also with the RLD, using the exact PSF used for blurring. Our algorithm converged with a mean squared error (MSE) of $2.92\times 10^{-11}a.u.$, whereas the RLD achieves a MSE of $8.76\times 10^{-13}a.u.$ The initial signal‐to‐noise ratio (SNR) of the synthetic measurement image over its background noise is 69.10 dB, whereas it increases to 80.28dB for the Blind‐AU reconstruction and to 82.89 dB for the RLD. Remarkably, our blind method gets very close to what found with standard RLD where, instead, the PSF is supposed to be known.

### 2.3 Multiview blind deconvolution of zebrafish samples

**FIGURE 4:**
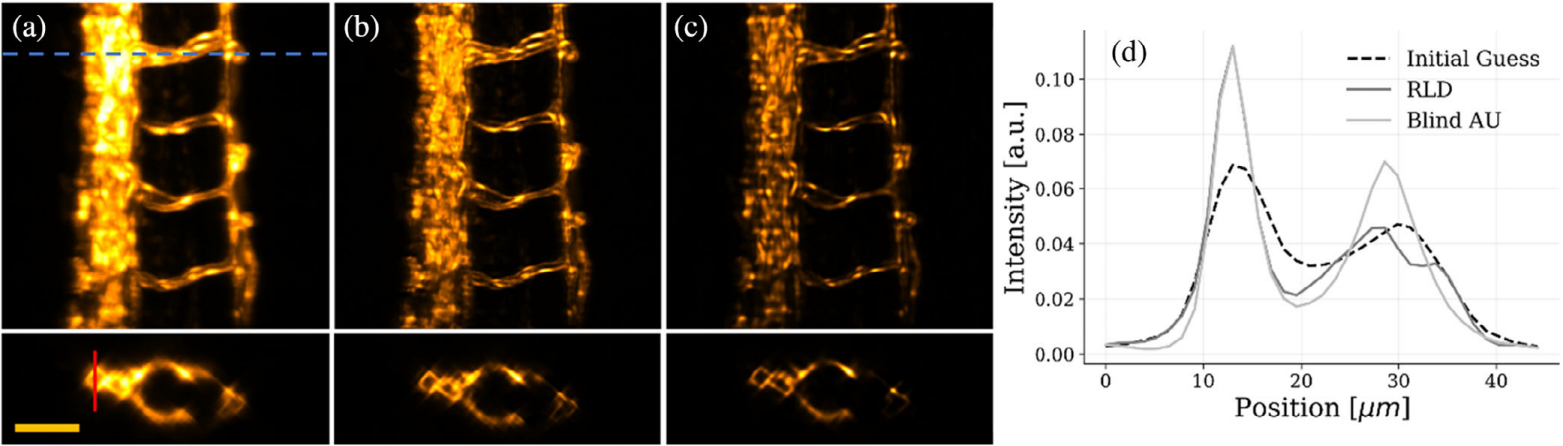
Four‐view RLD and blind‐AU reconstructions of the tail of a zebrafish embryo. (a) Top: Maximum intensity projection (MIP) of the fusion of four orthogonal views (45°, 135°, 225°, 315°) of the zebrafish tail section, used as the initial guess for RLD and blind‐AU algorithms. Bottom: Tomographic section of the same sample taken at the location of the dashed blue line. Scale‐bar: 50 μm. (b) MIP and tomographic section of the RLD reconstruction. (c) MIP and tomographic section of the reconstruction using the blind‐AU algorithm. (d) Intensity profile along the red line traced on the tomographic section (a) plotted for the initial guess, RLD, and blind‐AU reconstruction

After validating the method with synthetic samples, we tested it experimentally in a multiview light‐sheet microscope. We report the reconstruction results obtained with zebrafish (*Danio rerio*) embryos having the vasculature stained with a fluorescent protein. Experimental details are provided in the Materials and Methods section. For this experiment, we acquired four orthogonal views of the specimen, focusing on the central region of the tail. In experimental measurements, we do not have access to the ground truth reconstruction of the specimen, so we compare our reconstructions with the one provided by standard RLD by assuming known the blurring PSF. To obtain this reconstruction, we initially align each acquisition against the other by locating the position of the maximum within their cross‐correlation. Once aligned, we fuse the volumes by calculating their average. Figure 4a shows the maximum intensity projection (MIP) of the fused volume (upper panel) and a tomographic slice along the dashed blue line (bottom panel). We estimate the PSF of a single view by measuring fluorescent nanobeads, fitted with a three‐dimensional Gaussian function. Finally, the PSF that blurs the multiview reconstruction is obtained by rotating the single‐view PSFs accordingly to the acquisition angle of each dataset. We use the average PSF to enhance the resolution of the dataset via RLD; the resulting volume is shown in Figure 4b. For the Blind‐AU, we compute the average autocorrelation of each view, as described in the previous section. We recall that this step does not require any alignment process. Then, we feed this estimation to the algorithm described in Equations (1) and (2) and schematized in Figure 1. After running a total of $25\times 10^{4}$ iterations, we obtain the result displayed in Figure 4c. Our reconstruction is sharper and highly resolved compared to the standard fused data and to the deconvolved one (Figure 4a,b). In particular, we can appreciate the improvement along the tomographic plane (bottom panels of Figure 4), where our method neatly discriminates the two vessels located in opposite regions of the spine. To quantitatively assess the improvement, we examine the profiles of the three volumes along the direction indicated by the red line in Figure 4a. The plot of the profiles is shown in Figure 4d. As expected, the RLD gives results sharper than the initial guess. Our method further surpasses this result by better separating the opposite vessels, reconstructing details sharper than standard approaches. Respectively for RLD and Blind‐AU, the convergence MSE is $4.06\times 10^{-16}\;a.u.$ and $3.20\times 10^{-15}\;a.u$. The image quality of the direct measurement is quantified by the image SNR and is equal to 63.55dB. RLD brings the metric of the reconstruction to 72.01dB, which is further improved by the Blind‐AU algorithm to 79.93dB.

**FIGURE 5:**
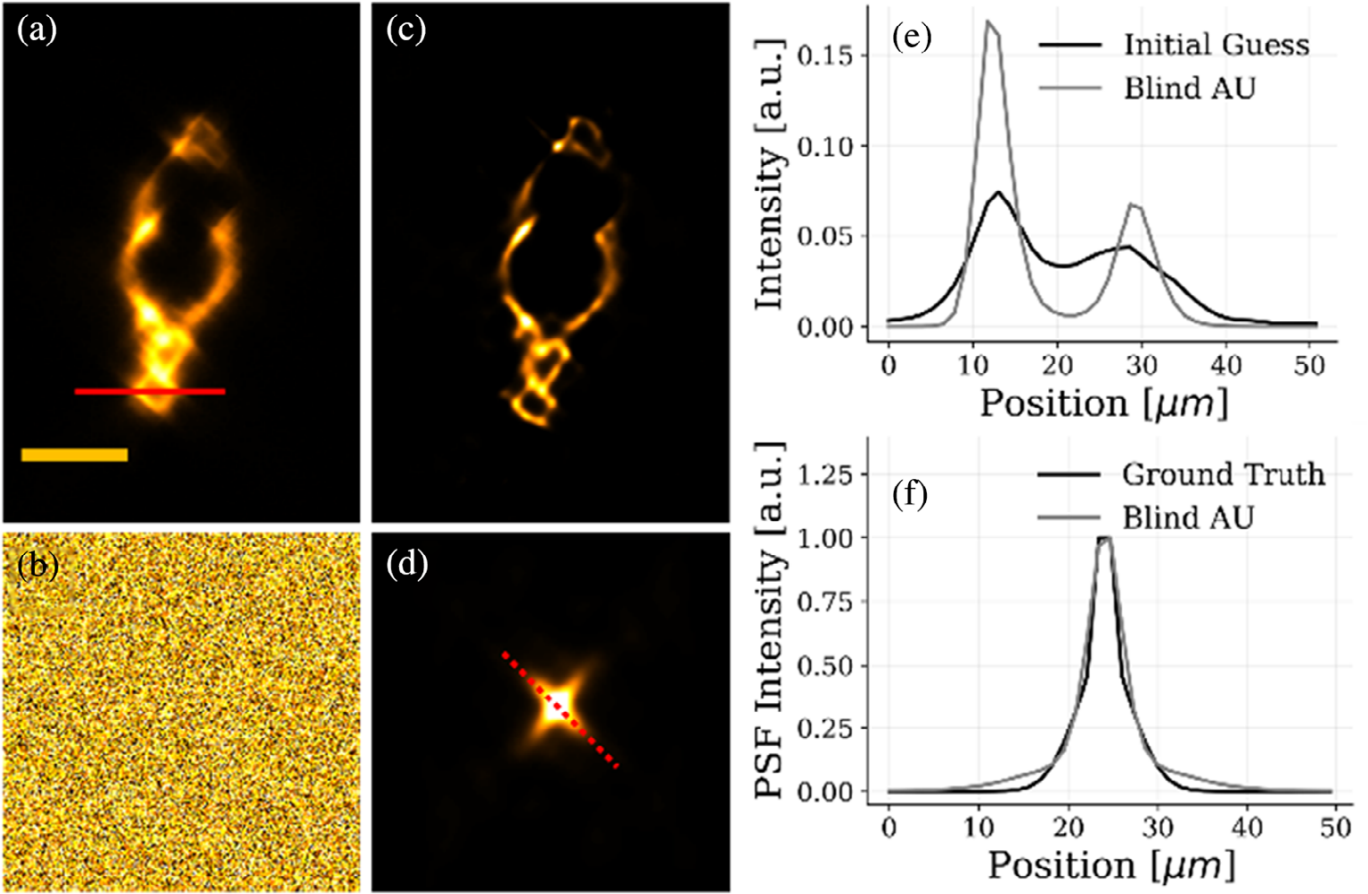
Multiview blind‐AU reconstruction of a sample slice starting with a random PSF initial guess. Blind‐AU algorithm applied on a tomographic selected slice of the four orthogonal views of the tail of a zebrafish embryo (45°, 135°, 225°, 315°). (a) Initial guess for the sample, corresponding to the aligned fusion of the four orthogonal views. (b) Random initial guess for the PSF. We assume to have null information about the structure of the PSF of our volume. (c) Tomographic slice reconstructed with our blind‐AU method. (d) the PSF obtained after the blind optimization routine. (e) Intensity profiles plotted along the red line traced in (a) for the sample initial guess and blind‐AU reconstruction. (f) Intensity profiles plotted along the red dashed line in (d) for the PSF ground truth and blind‐AU reconstruction

Having assessed the image reconstruction ability, we now focus on the quality of the PSF obtained at the end of the process. We take a slice along the tomographic plane obtained by fusing orthogonal views (Figure 5a). We first average the autocorrelations of all the views. The Blind‐AU is initialized with the fused object reconstruction and a completely random guess for the PSF (Figure 5b). The object reconstruction and PSF are optimized alternatively with our method. After running $5\times 10^{4}$ iterations, we obtain the reconstructions of the object displayed in Figure 5c, and of the PSF in Figure 5d. Also, in this case, the increase in the resolution can be verified by the intensity plot along the red line of panel a: our method reconstructs edges that are sharper than the original fused data (Figure 5e). The gain in resolution is made possible thanks to the recovery of an accurate PSF estimation, shown in Figure 5d. We notice that the recovered PSF is elongated towards orthogonal directions, as expected in the reconstructions based on multiview LSFM. We have previously estimated the elongation of the point spread function along the scanning axis (the worse resolution in the image); thus, we compare it with the profile along the elongated direction of the recovered PSF. The resulting graph is plotted in Figure 5f. Our method blindly recovers a PSF close to the measured one, guarantying a resolution increase even when completely ignoring the optical response of the system. Quantitatively, the Blind‐AU algorithm improves the image quality of the sample also in the case of a random PSF initial guess: starting from an image SNR of 64.47dB the value is increased to 76.77dB after the iterative process. In this case, we assume convergence when the MSE is $1.42\times 10^{-10}a.u$.

## 3 CONCLUSIONS

In this article, we have introduced a new method to form aligned reconstructions in multi‐view light‐sheet microscopy. Compared to the state‐of‐the‐art algorithms, our implementation obtains sharp and inherently aligned reconstructions in the case where we have limited knowledge of the optical response of the measuring setup. To achieve this, we have generalized the AU algorithm (Ancora & Bassi, 2021), which we used for alignment‐free multiview reconstruction imaging (Ancora et al., 2021), by including the blind deconvolution feature. Our choice was to alternate the reconstruction of the object with that of the point‐spread function, keeping one fixed while solving for the other. To this end, we took inspiration from blind strategies grounded on the Richardson‐Lucy deconvolution approach (Fish et al., 1995; Richardson, 1972) but here, instead, starting from the estimation of a global autocorrelation. We have already proven that autocorrelations can form implicitly aligned reconstructions down to the sub‐pixel level (Ancora et al., 2021); thus, we decided to extend further its range of applicability. Thanks to its design, our method forms accurate reconstructions from multi‐view acquisitions without knowing the PSF of the system. The validation was assessed under three different regimes and followed the actual software development. Initially, a blurred synthetic specimen was reconstructed by fusing multiviews in the autocorrelation domain, proving the fidelity of the reconstruction against the known ground truth. Once validated numerically, we tested it with experimental acquisitions of a three‐dimensional specimen. We examined the vasculature of a zebrafish, a well‐known model organism that permits us to spot the formation of eventual artifacts in the reconstructions. These reconstructions were compared with those obtained by standard deconvolution routines, demonstrating a contrast increase and robustness to experimental noise. Although we have not studied the effect of increased noise, a similar level of SNR for the synthetic (purely Poissonian) and experimental samples leads to comparable results. This is in line with what is commonly assumed in deconvolution works (Lucy, 1974; Richardson, 1972). On the other hand, additive noise accumulates in autocorrelation space as a delta centered in the origin (Ancora & Bassi, 2021) Then, since the effect of random intensity fluctuations in $\chi_{\mu }$ is localized, finding the correct value associated to the zero‐shift pixel could potentially provide denoised reconstructions. In the ultimate analysis, we have assessed the correct recovery of the PSF in our multiview LSFM setup, comparing it with the measured optical response along the scanning axis. The setup used in our experiment was a custom‐made light‐sheet fluorescence microscope with a rotating stage but, our technique is readily usable in any omnidirectional LSFM setups (Weber & Huisken, 2012), or in techniques directly based on autocorrelation inversion such as hidden tomography (Ancora et al., 2020).

Although it guarantees excellent reconstruction capabilities, our method may have room for further improvements in several aspects. Concerning the multiview aspect, the objects that appear sharp in one view may display less contrast compared to the view belonging to the opposite sides of the specimen. Even assuming the scattering to be negligible, the presence of tissue absorption weakens the signal coming from deep inside the tissue and makes the fusion problem non‐trivial. In turn, this implies that to consider the average of a view may not be the ideal choice to obtain the best reconstruction even if the datasets are perfectly aligned. If the datasets are misaligned (as it typically is), the problem gets even worse and binds to the fusion problem: the optimal fusion does depend upon prior alignment. As thoroughly discussed, our method avoids the alignment procedure by using autocorrelations, and we fuse the views by taking their average. A further in‐depth study on the assessment of the optimal choice for fusion might indeed be facilitated with our method, since fusion does not rely on alignment anymore. Our intention is to tackle this problem in follow‐up studies, thus, pushing the quality of autocorrelation‐based tomography further.

From a computational perspective, algorithms based on autocorrelation inversions, such as AU and Blind‐AU, make use of intense calculations. When working with large three‐dimensional volumes, carrying out numerical convolutions and correlations could be challenging. For this reason, we decided to write our Python library to exploit GPU computations, and all the operations are carried out in the Fourier domain using real FFTs implemented in CUDA via the CuPy library. The use of GPUs permits the reduction of computing time by orders of magnitudes and renders the three‐dimensional problem approachable.

In terms of the solution of the inverse problem, the AU algorithm is a slowly converging method (Ancora et al., 2021) which we iterate blindly for tens to hundreds of iterations within each cycle. Trying with a different number of blind iterations and selecting a careful choice of initial guesses may speed up the recovery process. Similar Bayesian approaches have already benefit enormous speed‐up by choosing ad‐hoc backpropagating kernels (Guo et al., 2020). This idea is suitable for our implementation and can lead to faster solutions. Since our formulation is structurally similar to RLD, regularizing the solution is an attainable refinement. Total variation (Laasmaa et al., 2011) and sparsity constrained (Shaked et al., 2011) are two choices we plan to incorporate in our formalism to further enhance the reconstructions. Including priors could also enhance the performance of our method. For example, in multiview geometry, the angle of rotation is generally known. Designing a functional form for the PSF that accounts for the angular rotation could ease the reconstruction process. Lastly, our algorithm assumes uniform PSF blurring thorough the entire imaged volume. Although this is often considered a good approximation, higher image quality can be reached when considering spatially anisoplanatic deconvolutions (Toader et al., 2021) or mixed optical aberration corrections (Furieri et al., 2022). These considerations fall beyond the scope of the present manuscript, which may open new paths for further studies.

## 4 MATERIALS AND METHODS

### 4.1 Synthetic sample generation and measurement

To test the Blind‐AU algorithm, we generated an image simulating blood vessels. The simulated multi‐view measurement is composed of two orthogonal views of the sample, generated by convolving the original image with an elongated Gaussian PSF (with standard deviation $\sigma_{z}$ = 2.7 px, $\sigma_{x}$= 1 px), oriented along two different axes ($\theta_{1}=0{}^{\circ}$ and $\theta_{2}=90{}^{\circ}$). Then, images are normalized to $2^{16}$ and Poisson noise is added, with average and variance equal to $\lambda =2^{8}$. For *N* different views, identified by the subscript $\theta_{i}$, each simulated measurement is determined by:

(3) $$f_{\mu ,\theta_{i}}=o*h_{\theta_{i}}+\epsilon_{\theta_{i}}$$

Where $h_{\theta_{i}}$ is the PSF rotated by the angle $\theta_{i}$, and $\epsilon_{\theta_{i}}$ is the Poisson noise added to every single view. Each matrix has an odd dimension of 191 × 191 pixels, so the rotation of the PSF is applied around the central pixel, and the Gaussian is perfectly symmetric with respect to the center of the image. The resulting $f_{\mu ,1}$ and $f_{\mu ,2}$ are displayed in Figure 2.

### 4.2 Light‐sheet fluorescence microscopy

Light‐sheet fluorescence microscopy is an optical technique that exploits decoupled illumination and detection, placed orthogonal to each other, to achieve optical sectioning. The illumination arm generates a light sheet (a single plane of illumination) on the sample. The emitted fluorescence is collected and filtered along the detection path, and forms an image on a widefield detector (CMOS camera). Having independent illumination and detection, the PSF is determined by the product between illumination and detection PSFs and is elongated, typically, along the axial direction (Power & Huisken, 2017). The system starts with a single‐mode blue laser emitting at 473nm (MBL‐FN‐473, 50mW power), operating in CW mode. The laser beam is collimated to a diameter of 4mm (Thorlabs collimator RC04FC‐P01), and its width is reduced to 1mm by a vertical slit. Then, the light is sent to a galvanometric mirror (Thorlabs GVS001) which is conjugated with the sample position along the optical illumination path. The galvanometric mirror is controlled by a sinusoidal voltage signal and oscillates with an amplitude of ±1° and frequency of 250Hz. This pivots the illumination beam to remove shadowing artifacts that are common for side illumination of the sample (Di Battista et al., 2019). Then, the beam is expanded by a factor of 3 by a telescope (4f system, with $f_{1}=50$ mm and $f_{2}=150\;\mathrm{mm}$), and a cylindrical lens (f=200mm) generates the light sheet, focusing light only along the vertical direction. The beam diameter is halved by a second 4f system ($f_{1}=400\;\mathrm{mm},f_{2}=200\;\mathrm{mm}$), and the light is collimated towards the back focal plane of the illumination objective (Olympus water immersion objective, 10×, NA=0.3). The emitted fluorescence is collected by a second identical objective, mounted in a perpendicular direction with respect to the illumination path. Then, the light crosses a GFP filter (center wavelength of 520 nm), the tube lens (f=180mm), and is collected by a low noise sCMOS camera (Neo 5.5 sCMOS Andor Technology). The size of the beam after the vertical slit results in an illumination numerical aperture $NA_{\mathrm{ill}}=0.0055$ and an axial resolution $\delta z_{\mathrm{ill}}=5.65\;\mathrm{\mu m}$, whereas the lateral resolution is $\delta \rho_{\det}=0.87\;\mathrm{\mu m}$. In the central unit of the microscope, the sample is mounted fixed to the mechanical support that controls its position and orientation using a translator (PI M‐403) and a rotator (PI M‐037). Volumes are acquired by scanning the sample through the illumination beam, with minimum exposure time for each slice (0.0101305s) and displacement along the detection axis between two consecutive acquisitions of 1.3μm. This value is also the lateral dimension of the region imaged by every single pixel, by applying a 2×2 binning to the camera.

### 4.3 PSF characterization

The point‐spread function (PSF), representing the intensity impulse response of the microscope, describes the image degradation process. The PSF was estimated experimentally by measuring a sample composed of sparse fluorescent nanobeads, much smaller than the microscope resolution. They act as individual point sources, whose recorded image describes the impulse response of the system. Beads should be measured under the same experimental conditions as the sample, to avoid variations due to wavelength changes, and retrieve information about the alignment of the optical elements (Sibarita, 2005). We used fluorescent nanobeads (Estapor, XC dye, 100 nm size) absorbing between 460 and 500nm and emitting in the 515−570nm range. They are embedded in phytogel with a concentration of $1:10^{5}$ and inserted in a cylindrical FEP tube with an internal diameter of 2mm and an external one of 4mm. To characterize the 3D PSF, we acquired an entire volume of beads at the same experimental conditions as the sample; then, we selected one fluorescent bead located in the center of the FOV and applied a Gaussian fit along the three dimensions to retrieve an analytical expression. The ground‐truth PSF of the 2D reconstruction (Figure 5) was estimated, instead, by fitting a bead located in the same region of the FOV as the sample slice. In general, the microscope PSF may vary when moving away from the center of the FOV, but the isoplanatic hypothesis can be considered valid in the portion of the FOV used in this work. In fact, experimental beads maintain a homogeneous shape and dimensions in the volumetric region occupied by the sample.

### 4.4 Zebrafish handling and preparation

Adult zebrafish were maintained according to National (Italian D.lgs 26/2014) and European laws (2010/63/EU and 86/609/EEC), controlling experiments on live animals. Embryos, collected by natural spawning, were staged and raised at 28°C in fish water (Instant Ocean, 0.1% methylene blue). We used zebrafish embryos (*Danio rerio*) belonging to the line Tg(fli1a:EGFP)y1. The entire vascular tree can be visualized in vivo, thanks to the expression of Enhanced Green Fluorescent Protein (EGFP) under the control of the endothelial‐specific gene promoter fli1a (Kimmel et al., 1995; Lawson & Weinstein, 2002). At 48 hours post‐fertilization, embryos have a typical dimension of $700\times 700\times 3000\;\mu m^{3}$. They are anesthetized and mounted in Fluorinated Ethylene Propylene (FEP) tubes (outer diameter of 1.6 mm, inner diameter of 0.8mm, 3cm long), filled with 0.1% agarose, and a final tricaine concentration of 160mg/L (Kaufmann et al., 2012). The tube is mounted in the microscope chamber, filled with a stock solution composed of fish water with 0.016% tricaine concentration.

### 4.5 Richardson–Lucy deconvolution

Richardson–Lucy deconvolution (RLD) algorithm is an iterative numerical method for image restoration, stable in presence of high noise levels (Lucy, 1974; Richardson, 1972). It is derived by interpreting images and PSFs as probabilities and applying Bayes theorem. In microscopy, the system's point spread function, h, is not ideal; thus, the measurement $f_{\mu }$ of an object o can be described by the convolution $f_{\mu }=o*h+\epsilon$. In this description, we can state that pixel‐value of $f_{\mu }$ is determined by the re‐distribution of the energy of o accordingly to h. Assuming that the PSF is shift‐invariant (isoplanatic condition), the RLD iterative process can be written in a convolutional form:

(4) $$o^{\left(t+1\right)}=o^{\left(t\right)}\left(\frac{f_{\mu }}{o^{\left(t\right)}*h}*\tilde{h}\right).$$

With the notation $\tilde{h}$, we indicate the PSF with reversed coordinates: $\tilde{h}=h\left(-x\right)$, and the index t identifies iterations. Starting from an initial guess $o^{0}\geq 0$, nonnegativity is preserved during the iteration process, without the need for positivity constraints (Fish et al., 1995).

### 4.6 Anchor‐update deconvolved deautocorrelation

Our blind method strongly relies on the Anchor‐update (AU) algorithm (Ancora & Bassi, 2021) that, here, we recall for completeness. The goal is to recover a sharp object $o_{\mathrm{rec}}$ from the measurement of the autocorrelation $\chi_{\mu }$ blurred by a given function H so that:

(5) $$\chi_{\mu }=\left(o_{\mathrm{rec}}⋆o_{\mathrm{rec}}\right)*H=\left(o_{\mathrm{rec}}*h\right)⋆\left(o_{\mathrm{rec}}*h\right).$$

Since in our specific case, H=h⋆h. The solution with respect to $o_{\mathrm{rec}}$ can be approached via an iterative fixed‐point multiplicative method similar to RL deconvolution:

(6) $$o^{t+1}=o^{t}\left[\left(\frac{\chi_{\mu }}{o^{t}*K^{t}}\right)*{\tilde{K}}^{t}\right],$$

where $K_{t}=o_{t}⋆H$ is an effective kernel continuously updated as the reconstruction progresses. Equation (6) defines the AU iteration as a function of the iteration step t.

### 4.7 Metrics to assess the image quality

We monitored the convergence trend by computing the mean squared error (MSE) that estimates the similarity between the final reconstruction blurred by the PSF of the system and the initial measurement:

(7) $$\mathrm{MSE}\left(s_{\mu }\|s\right)=\int {\left(o_{\mathrm{rec}}*h-s_{\mu }\right)}^{2}\;dx\;$$

Here we denote with $o_{\mathrm{rec}}$ the reconstruction obtained with the iterative algorithm, with h being the PSF of the system, and with $s_{\mu }$ the initial measurement. If this metric is applied to the RLD algorithm, h is the known PSF of the optical system. Instead, when considering the AU algorithm, h is the PSF estimated by the blind iterative process. The measurement $s_{\mu }$ and the blurred image $o_{\mathrm{rec}}*h$ are energy‐normalized.

On the other hand, we assess the image quality by calculating the signal‐to‐noise ratio (SNR) of the original measurements and the reconstructions, according to the expression:

(8) $$\mathrm{SNR}\left(s\|\epsilon \right)=20\log_{10}\left[\;\frac{s_{\max}}{\epsilon }\;\right]$$

where $s_{\max}$ is the peak intensity of the image, and ϵ is mean value of the background noise estimated by in a region of the image where the sample is not present.

The metrics defined in Equations (7) and (8) permit us to compare the convergence trend and reconstruction results among different algorithms and whether the iterative process provides a feasible reconstruction.

### 4.8 Preprocessing of experimental data

In the present manuscript, a multiview measurement is composed of four orthogonal views, acquired by rotating the sample by steps of 90°. First, we subtract the background signal from each matrix by measuring a full stack without laser illumination. Second, we rotate each stack against the reference view: the passage is performed in multiples of 90°. The measure passed to the algorithm lies in the autocorrelation space, and it is computed by autocorrelating each stack and averaging them. It guarantees intrinsic subpixel alignment (Ancora et al., 2021). Here, autocorrelation is carried out as multiplication in the Fourier domain, according to the cross‐correlation theorem. To avoid numerical errors before the fusion, we take the absolute value of each autocorrelation. To determine an initial guess for the sample image, we decided to align the views. This step is carried out by setting the first view as a reference, then computing the cross‐correlation between this stack and the others. For each couple, the position of the cross‐correlation maximum represents the translational shift to apply in the three spatial coordinates. Finally, the initial guess is computed as the average of all the aligned views. In addition, we removed all the null elements by substituting a small value, taken as 1/100 of the image average. Otherwise, those pixels will remain zero during deconvolution. It is worth noticing that the alignment procedure is not strictly necessary to run the algorithm but ensures convergence in case the object is truncated in the image plane.

## CONFLICT OF INTEREST

The authors declare that there is no conflict of interest that could be perceived as prejudicing the impartiality of the research reported.

## Data Availability

The data that support the findings of this study are available from the corresponding author upon reasonable request. The code to reproduce the results on synthetic samples is available on GitHub [18].
